# Toll-like Receptor 7/8 Agonists Exert Antitumor Effect in a Mouse Melanoma Model

**DOI:** 10.3390/medicina62010141

**Published:** 2026-01-09

**Authors:** Gheorghita Isvoranu, Mihaela Surcel, Ana-Maria Enciu, Adriana Narcisa Munteanu, Monica Neagu, Andrei Marian Niculae, Gabriela Chiritoiu, Cristian V. A. Munteanu, Marioara Chiritoiu-Butnaru

**Affiliations:** 1 ‘Victor Babeș’ National Institute of Pathology, 050096 Bucharest, Romania; mihaela.surcel@ivb.ro (M.S.); ana.enciu@ivb.ro (A.-M.E.); adriana.munteanu@ivb.ro (A.N.M.); monica.neagu@ivb.ro (M.N.); niculae.andrei@ivb.ro (A.M.N.); 2 Institute of Biochemistry of the Romanian Academy, 060031 Bucharest, Romania; gabi.chiritoiu@biochim.ro (G.C.); cristian.munteanu@biochim.ro (C.V.A.M.); mari.chiritoiu@biochim.ro (M.C.-B.)

**Keywords:** Toll-like receptors, imiquimod, gardiquimod, NK cells, melanoma, subcutaneous melanoma mouse model

## Abstract

*Background and Objectives*: Toll-like receptors (TLRs) are pattern recognition receptors with an essential role in regulating both the innate and adaptive immune response. Given their pleiotropic effects in mounting an immune response, previous studies have proposed targeting these TLRs might render alternative strategies for cancer therapy. Synthetic immune response modifiers, such as imidazoquinolines, stimulate the immune cells by activating Toll-like receptors, particularly TLR7/8 receptors, consequently mounting an immune response. Agonists of this class activate, via TLR-mediated signaling, dendritic and B cells, as well as myeloid cells and T cells, thus exhibiting good prospects for cancer immunotherapy. In the present study, we sought to evaluate the effect of imiquimod and gardiquimod, two TLR 7 and 7/8 agonists, respectively, on tumor growth and phenotype of NK cells associated with melanoma. *Materials and Methods*: We generated a syngeneic model of melanoma in C57BL/6J mice by subcutaneously injecting murine melanoma cells and monitoring tumor growth. Starting on day 8 or 14, we applied TLR agonists either intratumorally or topically and followed the tumor dynamics and NK cell-associated pattern. *Results*: Our results suggest that both TLR agonists displayed an antitumor effect along with a phenotypically activated profile of NK cells. Both imiquimod and gardiquimod treatment inhibited tumor growth, with gardiquimod showing an increased potency compared to imiquimod. *Conclusions*: This implies that TLR agonists like imiquimod and gardiquimod could serve as neoadjuvant, adjuvant, or complementary immunotherapeutic agents in melanoma therapy.

## 1. Introduction

Cutaneous melanoma is considered one of the most common forms of skin cancer, and its incidence is rapidly increasing. The Global Burden of Disease 2023, investigating causes of death, concluded that, between 2000 and 2023, the number of deaths due to malignant skin melanoma increased by 58.7% [1]. The plurifactorial nature of melanoma etiology, spanning from genetic predisposition to specific populational context, associated, for example, with high cumulative ultraviolet ray exposure, has led to the need to develop novel therapeutic strategies [2]. While a specific subset of tumors (such as NRAS-mutated tumors) can infer a specific etiology which is therapeutically addressable in preclinical models, the more heterogeneous, age-related, UV-exposure-augmented melanomas are still in need of novel therapeutic targets [3,4]. The immune system plays a major role in regulating tumor proliferation and initiating defense responses against tumor aggression. The resistance of melanoma to conventional therapies and its high immunogenicity justify the development of new therapies aimed at stimulating an effective immune response against melanoma [5]. Since recent studies and clinical trials have proven that immune cells can be efficiently directed to fight cancer cells with high specificity and lower side effects, multiple approaches to boost the immune response have been developed. Amongst these, NK cell-targeting therapies have increased considerably for cancer treatment.

The most recent findings on NK cell activation in cancer patients indicate that several important parameters, such as the ability of the tumor to modulate NK cell functions and phenotype, justify the development of NK cell-based therapies. NK cells can directly recognize conserved pathogen-associated molecular patterns (PAMPs) through Toll-like receptors (TLRs) and respond via cytokine production and NK-mediated cytotoxicity [6]. The direct recognition of pathogens by NK cells adds a new dimension to a cell whose activity is regulated not only by a balanced set of inhibitory and activating receptors but also by pattern recognition receptors (PRRs) [7]. NK cells express TLR-2, -3, -5, and -6, which recognize and respond to PAMPs, while TLR-4, -7, -8, and -9 are poorly expressed on these cells [8,9]. TLR-2 and TLR-3 agonists appear to act directly on NK cells, whilst TLR-7, -8, and -9 agonists, through interleukin (IL)-18 and IL-12 secreted by antigen-presenting cells (APCs), causing indirect NK cell activation translated by induction of interferon gamma (INF-γ), high levels of CD69, and increased antitumor cytotoxicity. Thus, TLR agonists represent a class of molecules with potential to activate both innate and adaptive immune responses to generate effective antitumor effects [10].

Imiquimod, a synthetic compound belonging to the imidazoquinoline family, is a TLR7 agonist approved in 1997 by the U.S. Food and Drug Administration for topical treatment of superficial basal cell carcinoma, actinic keratoses, and virus-induced skin neoplasms. In addition to skin cancer, numerous experimental studies have confirmed the antitumor role of imiquimod in other types of cancer, such as kidney cancer, intracranial tumors, and breast cancer, and it is occasionally used for melanoma treatment [11,12,13,14,15]. Topical administration of imiquimod was shown to increase the number of NK cells and DCs in the lungs in a non-cancerous mouse model [16]. Another member of the imidazoquinoline family is gardiquimod, a TLR7/8 agonist, with a ten-fold increased potency compared to imiquimod, shown to stimulate the activation of T and NK cells [17].

TLR7/8 agonists activate signaling pathways leading to the induction of interferons, proinflammatory cytokines, and chemokines, and the main benefit of these agonists is that in addition to activating antigen-presenting cells, they also promote activation of cytotoxic T and NK cells [14,18,19]. Imiquimod, through its TLR7 agonist activity, displays antiviral and antitumor potential, and topical application of imiquimod (ALDARA cream) in both humans and mice induced a strong inflammatory response, characterized, amongst others, by the activation of immune cells. Although the efficacy of imiquimod in the treatment of tumors is well documented, little is known about the effects of gardiquimod on tumors.

NK cells can be activated directly by TLR agonists or indirectly through cytokines released by dendritic cells and macrophages [17,20,21,22,23]. Dendritic cells and macrophages secrete cytokines such as IL-12, IL-15, IL-18, and type I IFN that are crucial for the activation of NK cells, which in turn via production of IFN-γ and tumor necrosis factor alpha (TNF-α) activate dendritic cells and macrophages [6]. Our previous results have shown that imiquimod administration to mice induced a significant increase in the blood level of numerous pro-inflammatory cytokines, including IL-12 (p70), IL-15, IFN-γ, and TNF-α [24].

In this study, we investigated the effects of two TLR 7 and 7/8 agonists, imiquimod and gardiquimod, respectively, on murine melanoma tumor growth and the phenotypic characteristics of splenic NK cells. Our results showed that the TLR agonists delayed tumor growth, an observation which could be used to choose an appropriate therapy against cutaneous melanoma.

## 2. Materials and Methods

### 2.1. Mice and Cell Line

C57BL/6J mice were purchased from The Jackson Laboratory (Bar Harbor, ME, USA) and bred in the animal facility of the “Victor Babeș” National Institute of Pathology, Bucharest, Romania. Mice, female and male, 8 to 10 weeks of age at the onset of the experiments, were used. The mice were housed under controlled conditions of light (12 h light/12 h dark), temperature (22 ± 2 °C), and humidity (55 ± 10%), and were given granulated food and water ad libitum. The experimental protocols were performed in accordance with Directive 2010/63/EU on the protection of animals used for scientific purposes and Romanian national regulations, and were reviewed and approved by the Ethics Committee of “Victor Babeș” National Institute of Pathology, Bucharest, Romania (No. 55/20.02.2018), and by the National Veterinary Sanitary and Food Safety Authority (No. 388/22.03.2018).

B16-F10 melanoma cells (ATCC^®^ CRL-6475™; ATCC, Manassas, VA, USA) were cultured in complete DMEM with 4,5 g/L glucose (Gibco, Life Technologies Limited, Paisley, UK) supplemented with 10% FBS (Gibco, Life Technologies Limited, Paisley, UK) and 1% penicillin-streptomycin (Lonza, Verviers, Belgium).

### 2.2. Chemicals

The TLR 7 agonists used in this study were a 5% imiquimod cream (ALDARA^TM^ 5%, Viatris Healthcare Limited, Dublin, Ireland), and powdered imiquimod (IMQ) and gardiquimod (GDQ), a TLR7/8 agonist, both obtained from InvivoGen (InvivoGen Europe, Toulouse, France). After the development of tumors, the treatment was administrated in two ways, topically and intratumorally.

### 2.3. Mouse Model of Subcutaneous Melanoma

On day one, prior to cancer cell inoculation, the back flank regions of the mice were shaved. B16-F10 melanoma cells were resuspended in phosphate-buffered saline (PBS, Gibco, Life Technologies Corporation, Grand Island, NE, USA) and injected subcutaneously (s.c.) into the right flanks of mice at 0.5 × 10^6^ cells per 100 µL using a 25 G needle.

On day 8 after tumor cell inoculation, mice were randomly allocated to study groups with similar tumor volumes (average across studies 32 mm^3^–92 mm^3^): ALDARA-treated mice (ALD), imiquimod-treated mice (IMQ), gardiquimod-treated mice (GDQ), PBS-treated mice, and untreated melanoma-bearing mice (MbM) (*n* = 5 per group). Randomization occurred in a blinded fashion and the allocation remained concealed to the investigators that performed the flow cytometry analyses.

Development and growth of the tumors were monitored by measuring the tumor volume once or twice a week using a caliper. The volume of the tumors was calculated according to following formula: L × W^2^/2 (W = shorter diameter, L = longer diameter of the tumor).

### 2.4. In Vivo Treatment with TLR 7/8 Agonists

Topical treatment was applied starting on day 14, for 5 consecutive days. Tumor sites were treated with topical application of 83 mg of ALDARA cream. Intratumoral treatment was applied on days 8, 10, 15, and 18 by direct injection of IMQ or GDQ at a concentration of 1 mg/kg, as these doses have been shown to be safe and effective for antitumor therapy and, in parallel, the control group received PBS [17,25,26]. All mice were monitored daily, and tumors were measured by two independent and experienced investigators according to the above-described procedure to estimate treatment efficiency on melanoma growth. Except for one mouse from the GDQ group which presented tumor regression, all mice were euthanized on day 21 along with the control group—healthy mice (*n* = 5).

### 2.5. Flow Cytometric Analyses of NK Cells

Spleens were harvested from euthanized mice and processed to obtain single cells using a previously described protocol [27]. Briefly, spleen cell suspensions were resuspended in Cell Staining Buffer and incubated with TruStain fcX (anti-mouse CD16/32) antibody to block non-specific antibody binding (BioLegend, San Diego, CA, USA). Subsequently, the samples were stained with the following monoclonal antibodies: FITC anti-mouse CD3ε (clone 145-2C11), BV 510 anti-mouse NK1.1 (clone PK136), PE/Cy7 anti-mouse CD69 (clone H1.2F3), APC/Cy7 anti-mouse CD45R (B220) (clone RA3-6B2), PerCP/Cy5.5 anti-mouse CD11c (clone N418), PE/Cy7 anti-mouse CD335 (NKp46) (clone 29A1.4), APC/Cy7 anti-mouse CD43 (clone 1B11), PerCP/Cy5.5 anti-mouse/rat/human CD27 (clone LG.3A10), PerCP/Cy5.5 anti-mouse CD122 (IL-2R/ IL-15Rβ) (clone TM-β1), and Alexa Fluor 647 anti-mouse CD85k (gp49 Receptor) (clone H1.1) (all from BioLegend), and eFluor 450 anti-mouse CD49b (DX5) (clone DX5), APC anti-mouse CD11b (clone M1/70), and PE anti-mouse KLRG1 (clone 2F1) (all from eBioscience Inc., San Diego, CA, USA).

Unlabeled cells were used as controls; nonspecific fluorescence signals due to spectral overlap were automatically compensated (UltraComp eBeads; Thermo Fischer Scientific, Inc., San Diego, CA, USA). Data acquisition and analysis were performed on a BD FACSCanto II cytometer with BD FACSDiva v.6.1 software (BD Biosciences San Jose, CA, USA).

### 2.6. Statistical Analysis

The statistical software GraphPad Prism version 10 (San Diego, CA, USA) was used for data analyses. Data were presented as mean ± standard deviation of the mean (SD). Unpaired *t* test with Welch’s correction was used to compare tumor growth between different groups. One-way analysis of variance (ANOVA) with Tukey’s multiple comparison test was used to determine the statistical significance of differences between groups for NK cell investigation. Data were considered statistically significant at *p* < 0.05 (* *p* < 0.05, significant; ** *p* < 0.01, very significant; *** *p* < 0.001, highly significant; **** *p* < 0.0001, extremely significant).

## 3. Results

### 3.1. Topical Administration of a TLR 7 Agonist (Imiquimod)

**Figure 1:**
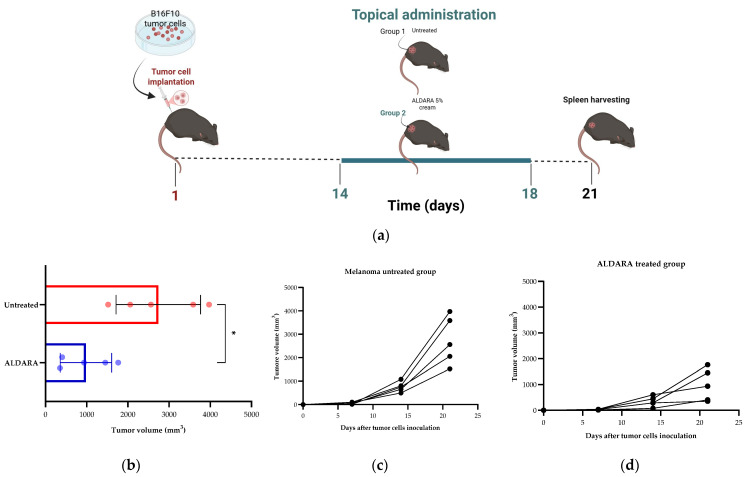
Effect of topical treatment with imiquimod (ALDARA cream) on melanoma. (**a**) Schematic diagram of the experimental protocol used to determine the efficacy of imiquimod (ALDARA cream) as a topical immunotherapy for melanoma. On day one, B16-F10 cells were implanted subcutaneously into the right flank of C57BL/6 mice. On day 14, for 5 consecutive days, topical treatment with 5% imiquimod cream (ALDARA) was applied to the tumor sites. Green line marks the treatment time. Schematic diagram of the experimental protocol was created at Biorender.com. (**b**) The endpoint tumor volume (mm^3^) in each group is represented graphically. (**c**,**d**) Tumor volume (mm^3^) measurements in untreated melanoma group and ALDRA-treated group (each line represents one mouse). Unpaired *t* test with Welch’s correction was used to compare tumor growth between groups (*n* = 5 per group). Data are presented as mean ± SD; * *p* < 0.05.

To evaluate the efficacy of topical administration of imiquimod, mice were inoculated subcutaneously into the right flank with B16-F10 cells on day 1, and after development of tumors, ALDARA cream was topically administrated, starting on day 14, for 5 consecutive days (Figure 1a). We observed that, at 21 days post-transplantation, the tumors of mice in the control, untreated group doubled in dimension compared to day 14, while treatment with ALDARA restrained tumor growth (Figure 1b–d). When comparing the tumor size (mm^3^), we found that the tumors in the ALD group were significantly lower than those in the control group (*p* < 0.05) (Figure 1b).

**Figure 2:**
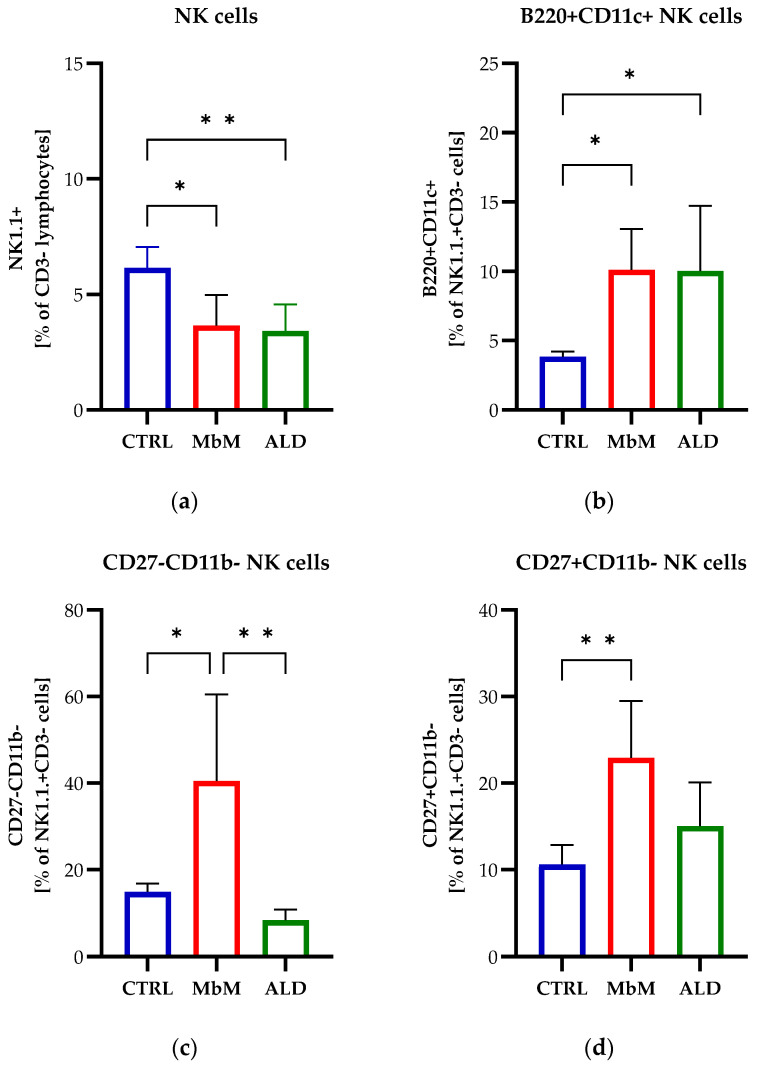

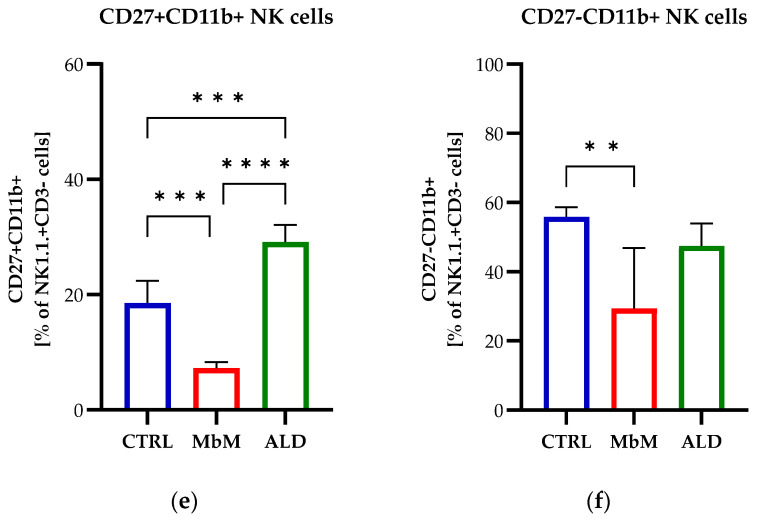
Flow cytometric analyses of total NK cells and NK cell subpopulations from spleen of C57BL/6 mice (CTRL) vs. melanoma-bearing mice (MbM) and melanoma-bearing mice treated with ALDARA (ALD). Percentages of total NK cells from lymphocytes CD3- (**a**). Percentages of NK cell subpopulations with B220 and CD11c surface expression (**b**) and different maturation surface expression profiles of CD11b and CD27 (**c**–**f**). One-way ANOVA with Tukey’s multiple comparisons test was used to determine the statistical significance differences between groups (*n* = 5 per group). Data are presented as mean ± SD; * *p* < 0.05, ** *p* < 0.01, *** *p* < 0.001 and **** *p* < 0.0001.

We hypothesized that topical administration of imiquimod could affect NK expansion; thus, we analyzed the NK cell fraction isolated from the spleen of mice used for experiments. B220 (CD45R) and CD11c are cell surface proteins primarily expressed on B cells, with B220 serving as a common marker for B cells in mice, or dendritic cells, but they are also found on other immune cells, including NK cells. Resting mouse NK cells express low amounts of B220 or CD11c, which become highly upregulated after activation with cytokines or other pharmaceutical agents, both *in vitro* and *in vivo* [28,29,30]. These proteins behave as classical activation markers on NK cells, with B220+CD11c+NK1.1+ cells having the capacity to produce considerable amounts of cytokines [31]. When analyzing the NK cell percentage in the spleen, we observed a significant decrease in the percentage of NK1.1+ cells in both groups, ALDARA (*p* < 0.05) and melanoma (*p* < 0.01), compared to healthy mice (Figure 2a). Conversely, B220 and CD11c expression on NK cells showed a significant increase in B220+CD11c+NK1.1+ cells for the melanoma groups (*p* < 0.05), with or without treatment, compared to healthy mice (Figure 2b). This subset of NK cells has been shown to exhibit functional characteristics similar to human CD56bright cells [31].

We also monitored the levels of CD27 and CD11b which have been considered as markers of NK cell maturation [32]. CD27 is a co-stimulatory receptor, constitutively expressed on T cells, B cells and NK cells, and its expression increases after cytokine stimulation while the CD27/CD70 interaction induce activation and enhanced immune response [33,34]. CD11b is a surface protein particularly expressed in the myeloid lineage, as well as on NK cells [35]. CD27 and CD11b expression on NK cells defines four phenotypically and functionally different subsets, CD27- CD11b-, CD27+ CD11b-, CD27+ CD11b+, and CD27- CD11b+ cells [36]. The mature subsets exhibit distinct functions, such as cytokine-producing regulators, CD27+ CD11b+ cells, and potent cytotoxic effectors, CD27-CD11b+ cells. CD27-CD11b- cells are the most immature cells with impaired cytotoxic activity and limited capacity for cytokine production. The presence of these immature NK cells in the tumor microenvironment was associated with the progression of lung carcinoma [37]. Analysis of NK cell subsets, based on the cell surface expression profiles of CD11b and CD27, indicated a significant decrease in mature subsets, CD27+CD11b+ (*p* < 0.001) and CD27-CD11b+ (*p* < 0.01), and an increase in the immature NK cell subsets, such as CD27-CD11b- (*p* < 0.05) and CD27+CD11b- (*p* < 0.01), for the melanoma-bearing mice (MbM) group compared to the control group. ALDARA treatment induced a significant increase in the CD27+CD11b+ subset compared to MbM (*p* < 0.0001) and CTRL (*p* < 0.001) groups, as well as a significant decrease in the CD27-CD11b- subset compared to MbM (*p* < 0.01) (Figure 2c–f).

Considering the changes observed when evaluating the percentage of NK cells and NK cell subpopulations, we next aimed to evaluate the effect of ALDARA treatment on the expression of various NK cell receptors, as well as maturation markers for the splenocyte suspension. In this regard, we chose to analyze the expression of both activator and inhibitory NK cell surface markers such as CD69, NKp46, CD122, CD11b, CD43, KLRG1, and CD27. CD69 is an early activation marker and a costimulatory receptor on activated NK cells, and it is involved in the regulation of their immune function [38]. High CD69 levels are associated with poor prognosis in cancer [39,40]. NKp46 is a major activating receptor on NK cells, mediating recognition and control of tumor cells by triggering cytotoxic responses [41]. CD122 (IL-2/IL-15R β-chain) expression indicates the commitment of cells towards the NK cell lineage, with IL-15 being a critical cytokine for NK cell survival, proliferation, and differentiation [42,43]. NK cell maturation occurs gradually, involving the acquisition and loss of cell surface markers. CD49b (DX5) expression defines the early mature stage NK cells, followed by the acquisition of CD11b and CD43, mature NK cells capable to produce large amounts of IFN-γ. In the terminal mature stages, the downregulation of CD27 and upregulation of KLRG1 are associated with increased cytotoxic function [44,45]. gp49 receptor, also known as CD85K or LILRB4, is an inhibitory receptor belonging to the Ig superfamily-related receptors, being expressed on activated NK cells [46]. It can promote immune evasion and support tumor growth [47,48]. gp49R receptor and B220 presented reduced values compare to untreated mice, but still higher than healthy mice, while CD49b presented slightly increased values.

**Figure 3:**
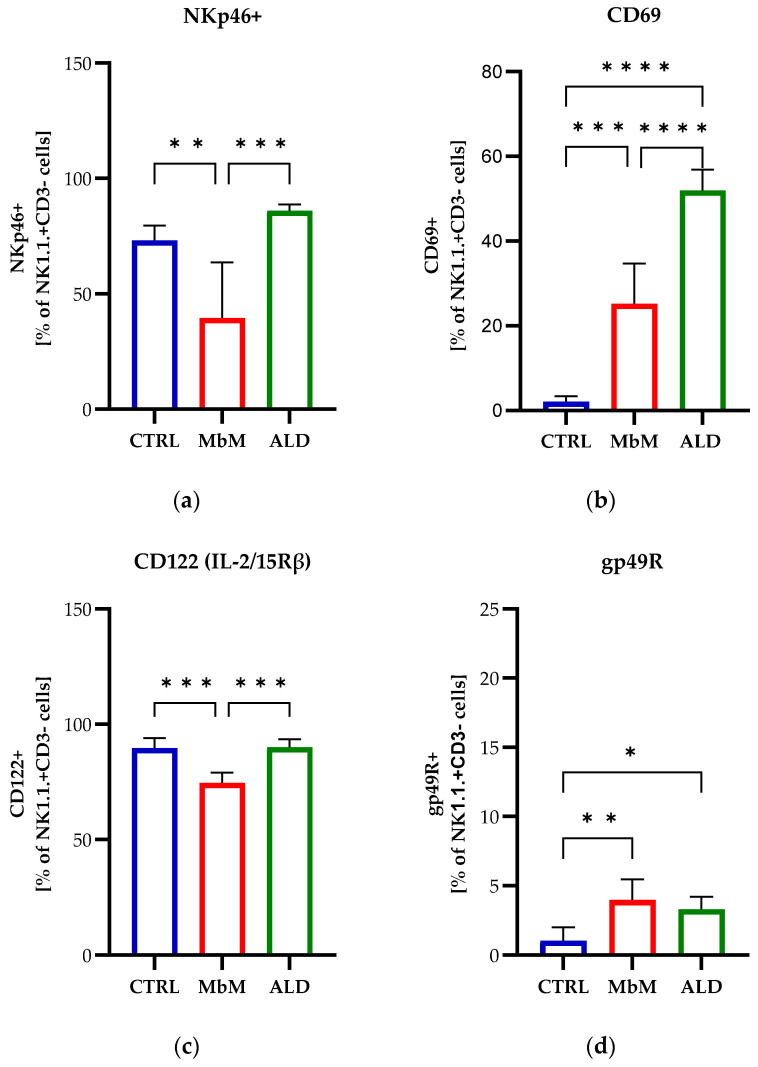
NK cells of C57BL/6 mice (CTRL) vs. melanoma-bearing mice (MbM) and melanoma-bearing mice treated with ALDARA (ALD) were analyzed by flow cytometry to estimate the expression of the activating receptor NKp46 (**a**), the activation-associated receptor CD69 (**b**), the co-activating receptor CD122 (**c**), and the inhibitory receptor gp49R (**d**). One-way ANOVA with Tukey’s multiple comparison test was used to determine the statistical significance of differences between groups (*n* = 5 per group). Data are presented as mean ± SD; * *p* < 0.05, ** *p* < 0.01, *** *p* < 0.001 and **** *p* < 0.0001.

Our results showed a decrease in the expression of the activating receptor NKp46 in melanoma-bearing mice (*p* < 0.01) compared to healthy mice, while the ALDARA treatment induced an increase in NK1.1+NKp46+ cells compared to the MbM group (*p* < 0.001) (Figure 3a). Conversely, the CD69 receptor, an early activation marker, was highly expressed in the melanoma group compared to the control group (*p* < 0.001) and further increased in the ALDARA-treated samples compared to both groups (*p* < 0.0001) (Figure 3b). When analyzing the expression on NK cells of the co-activating receptor CD122 (IL-2/15Rβ), we observed a decrease in the expression of this receptor for the melanoma group compared to the CTRL and ALD groups (*p* < 0.001); in mice treated with ALDARA, this receptor showed expression levels similar to those of the healthy group (Figure 3c). Also, tumor formation induced an increase in the percentage of NK cells expressing the inhibitory receptor gp49R (*p* < 0.01), and the administration of ALDARA led to a slight decrease in NK1.1+gp49R+ cells compared to the melanoma group, but the frequency of NK cells expressing gp49R remained significantly higher in ALD mice compared to the CTRL group (*p* < 0.05) (Figure 3d).

**Figure 4:**
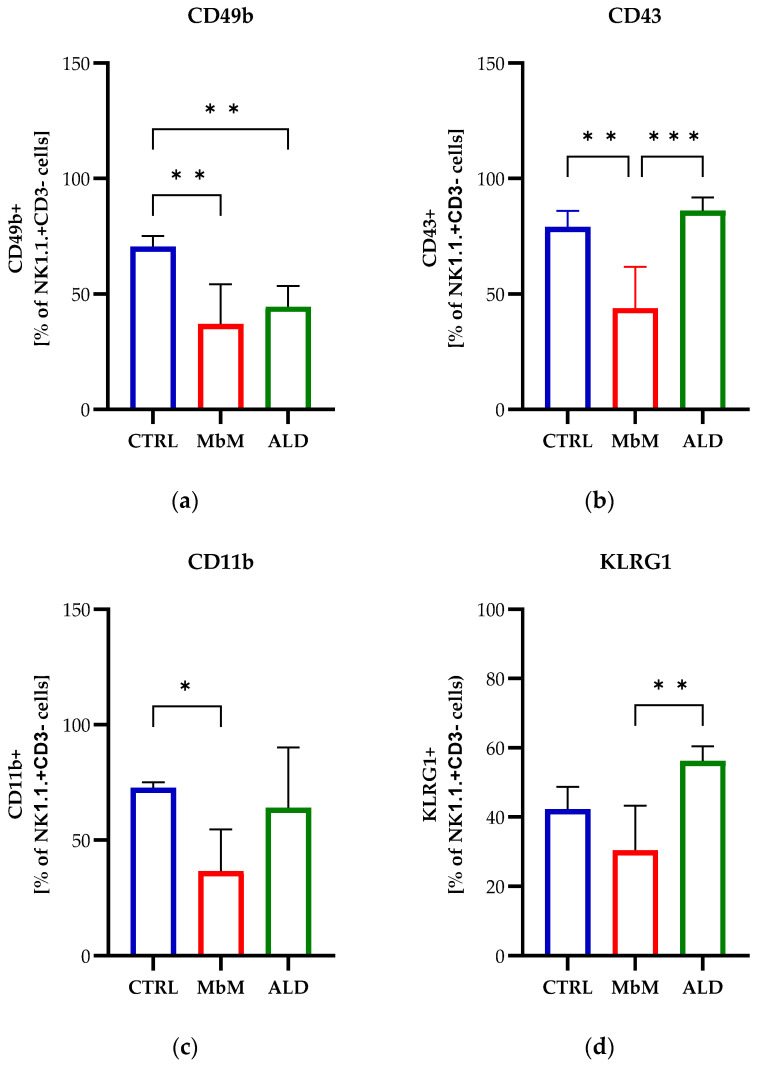

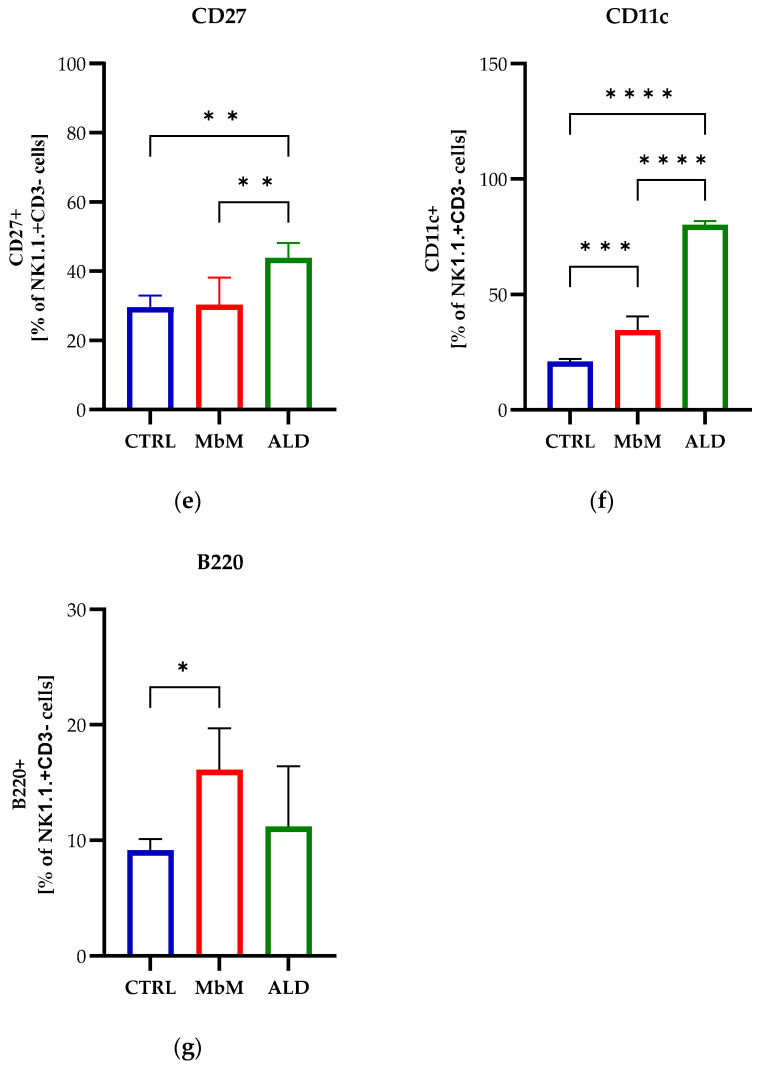
NK cells isolated from the spleen of control (CTRL) vs. melanoma-bearing mice (MbM) and melanoma-bearing mice treated with ALDARA (ALD) were analyzed by flow cytometry to quantify the endpoint expression of the maturation receptors CD49b (**a**), CD43 (**b**), CD11b (**c**), KLRG1 (**d**), CD27 (**e**), CD11c (**f**), and B220 (**g**). One-way ANOVA with Tukey’s multiple comparison test was used to determine the statistical significance of differences between groups (*n* = 5 per group). Data are presented as mean ± SD; * *p* < 0.05, ** *p* < 0.01, *** *p* < 0.001 and **** *p* < 0.0001.

When evaluating the expression of the maturation marker CD49b on NK cells, we observed a decrease in the NK1.1+CD49b+ cell percentage in both melanoma-bearing mice groups (*p* < 0.01), MbM and ALD, compared to the CTRL group (Figure 4a). Although the expression of the maturation markers CD43 and CD11b was decreased in the melanoma group (*p* < 0.01, and *p* < 0.05, respectively) compared to CTRL, the percentage of NK cells expressing these markers was increased for the ALD group compared to the MbM group, significant just for CD43 (*p* < 0.001), with values close to the CTRL group (Figure 4b,c). We also monitored the expression of KLRG1, which was higher for NK cells in the ALD group compared to the MbM group (*p* < 0.01) (Figure 4d). ALDARA treatment induced a significant increase in the percentage of NK cells expressing CD27 (*p* < 0.01) and CD11c (*p* < 0.0001) compared to CTRL and MbM mice, while a reduced frequency of NK cells expressing B220 could be observed in ALD mice compared to MbM mice, but increased compared to CTRL mice (Figure 4e–g).

### 3.2. Intratumoral Administration of TLR 7/8 Agonists

**Figure 5:**
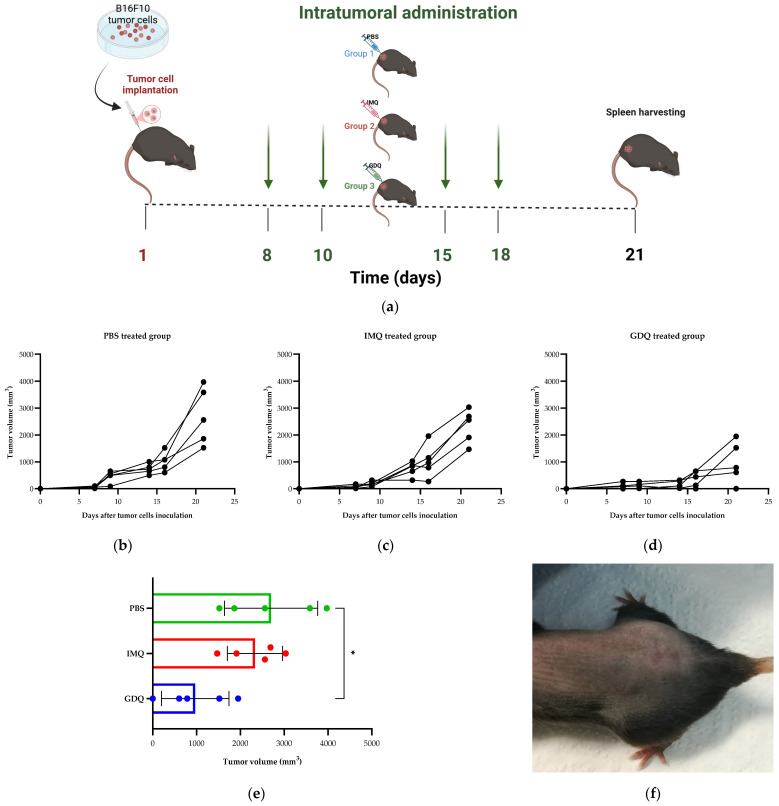
Effect of intratumoral treatment with imiquimod (IMQ) and gardiquimod (GMQ) on melanoma. (**a**) Schematic diagram of the experimental protocol used to determine the efficacy of IMQ and GMQ as an intratumorally administered immunotherapy for melanoma. On day one, B16-F10 cells were implanted subcutaneously into the right flank of C57BL/J6 mice. On days 8, 10, 15, and 18, intratumoral treatments with IMQ, GMQ, or PBS (control) were applied to the tumor sites. Green arrows mark the treatment time points. Schematic diagram of the experimental protocol was generated with BioRender. (**b**–**d**) Tumor volume (mm^3^) measurements in each treatment group represented as time-dependent variable (each line represents one mouse). (**e**) The endpoint tumor volume (mm^3^) in each treatment group. (**f**) Image of the mouse that received treatment with GDQ and shows tumor regression. Image was taken one month after the injection of tumor cells. Unpaired *t* test with Welch’s correction was used to compare tumor growth between groups (*n* = 5 per group). Data are presented as mean ± SD; * *p* < 0.05.

In the second experimental model, we evaluated the effect of IMQ and GDQ on melanoma development after intratumoral administration compared to the melanoma control group (PBS) and healthy mice. For this, B16-F10 cells were inoculated subcutaneously into the right flank of mice on day 1. After the development of tumors, on days 8, 10, 15, and 18, mice received treatment with IMQ, GDQ, or PBS, and were euthanized on day 21 (Figure 5a). In this experimental setup, we monitored tumor volume for each mouse and represented the values for each mouse from each group on separate graphs. The most prominent results observed when monitoring tumor size were observed for GDQ treatment, which led to a considerable inhibition of tumor growth compared to PBS treatment (*p* < 0.05), while IMQ administration showed an overall reduction in tumor volume, which was not statistically significant (Figure 5b–e). Of note, for one female from the GDQ group, tumor regression was observed after the first two administrations, and it appeared to be tumor-free on day 21 (Figure 5f). Since this was an atypical observation, the female was not euthanized on day 21, keeping track of tumor evolution, and after two months of monitoring, tumor re-growth was observed. At necropsy, on day 102, macroscopically visible melanoma metastases were observed on the lungs.

**Figure 6:**
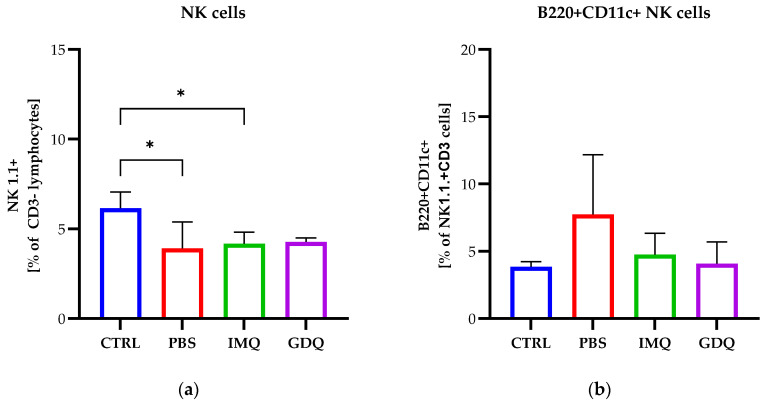

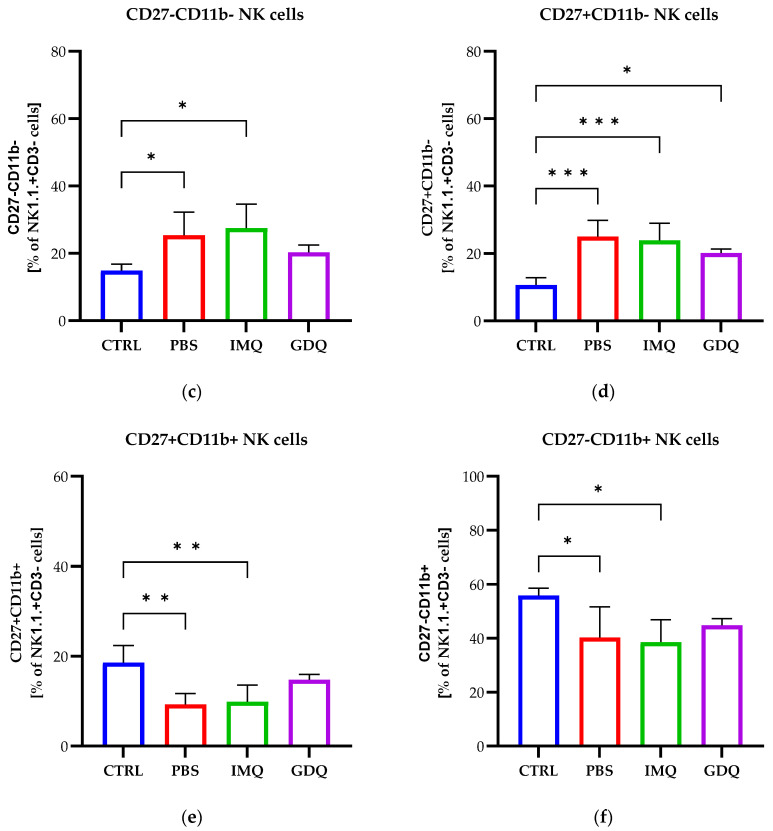
Flow cytometric analyses of total NK cells and NK cell subpopulations isolated from the spleen of C57BL/6 mice (CTRL, *n* = 5), melanoma-bearing mice (PBS, *n* = 5), and MbM treated with imiquimod (IMQ, *n* = 5) and gardiquimod (GDQ, *n* = 4). Percentages of total NK cells from lymphocytes CD3- (**a**). Percentages of NK cell subpopulations with B220 and CD11c surface expression (**b**) and different maturation surface expression profiles of CD11b and CD27 (**c**–**f**). One-way ANOVA with Tukey’s multiple comparison test was used to determine the statistical significance of differences between groups. Data are presented as mean ± SD; * *p* < 0.05, ** *p* < 0.01 and *** *p* < 0.001.

As we have previously stated, we were interested in evaluating the NK cell phenotype in mice treated with the two chemicals after melanoma induction. Thus, mice were euthanized on day 21 and spleens were processed to isolate NK cells. Similarly to the previously described experiments, when we used the ALDARA cream as topical therapy for melanoma, here we aimed to monitor the same NK cell populations and subsets. We analyzed the NK1.1+ cell population and observed no considerable differences between the treated groups and the PBS group, only a decrease for all melanoma-bearing mice compared with healthy mice, which was significant for the PBS and IMQ groups (*p* < 0.05) (Figure 6a). Next, we analyzed the percentage of B220+CD11c+NK1.1+ cells and our results showed that the IMQ and GDQ treatments resulted in a decrease in these cells compared to the PBS group, with values resembling those of the control group (Figure 6b).

Also, the analysis of NK cell subsets revealed an increase in the immature CD27-CD11b- NK cell subset in the PBS and IMQ groups compared to the control group (*p* < 0.05), while GDQ treatment induced an increase in CD27-CD11b- NK cell subset; however, this was not significant (Figure 6c). The CD27+CD11b- NK subset of cells was increased in all three melanoma groups compared to healthy mice (PBS and IMQ, *p* < 0.001; and GDQ, *p* < 0.05, respectively) (Figure 6d). The PBS and IMQ groups presented significant decrease in the mature subsets, CD27+CD11b+ (*p* < 0.01) and CD27-CD11b+ (*p* < 0.05), compared to the CTRL group (Figure 6e,f). GDQ also presented a decreased trend of mature subsets, but without statistical significance (Figure 6e,f). Treatment with IMQ did not induce significant changes compared to the PBS group, with mean values being relatively close to those of the PBS group. In contrast, GDQ treatment induced a slight decrease in the percentage of immature subsets and an increase in mature subsets compared to the vehicle control group (PBS).

**Figure 7:**
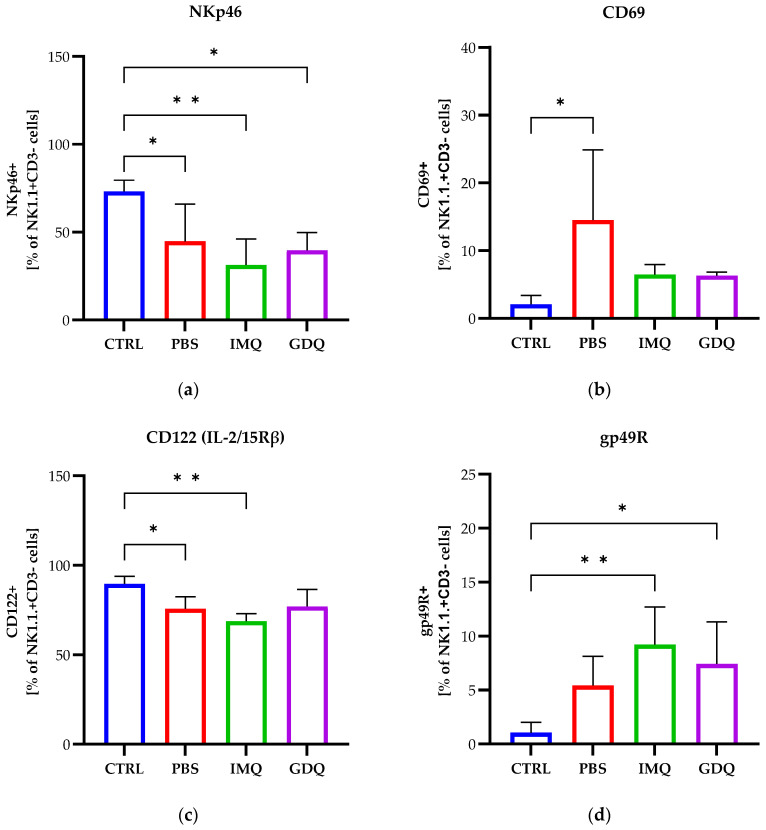
NK cells isolated from the spleen of C57BL/6 mice (CTRL, *n* = 5), MbM (PBS, *n* = 5), and MbM and treated with imiquimod (IMQ, *n* = 5) and gardiquimod (GDQ, *n* = 4) were analyzed by flow cytometry to determine the expression of the activating receptor NKp46 (**a**), the activation-associated receptor CD69 (**b**), the co-activating receptor CD122 (**c**), and the inhibitory receptor gp49R (**d**). One-way ANOVA with Tukey’s multiple comparison test was used to determine the statistical significance of differences between groups. Data are presented as mean ± SD; * *p* < 0.05 and ** *p* < 0.01.

In the next experiments, we aimed to monitor the cell surface expression of activation or inhibition makers for NK cells, as described above. For all three groups of melanoma-bearing mice, regardless of TLR 7/8 agonist treatment, we observed a significant decrease in the expression of the activating receptor NKp46 compared to healthy mice (PBS, *p* < 0.05; IMQ, *p* < 0.01; GDQ, *p* < 0.05) (Figure 7a), but no significant differences were observed after treatment with the indicated agonists compared to the vehicle control (PBS). An increased expression of the early NK cell activation marker, the CD69 receptor, was observed for the PBS group (*p* < 0.05) compared to healthy controls, which was reduced for both treatment groups but still higher compared to the healthy mouse group (Figure 7b). Conversely, a decreased expression of the co-activating receptor IL-2/15Rβ was observed, with more prominent differences for the PBS and IMQ groups (PBS, *p* < 0.02; IMQ, *p* < 0.01) (Figure 7c). The percentage of NK cells expressing the inhibitory receptor gp49R was increased in all three groups compared to the control group, with higher differences observed for the IMQ and GDQ groups (*p* < 0.01, *p* < 0.05, respectively) (Figure 7d).

**Figure 8:**
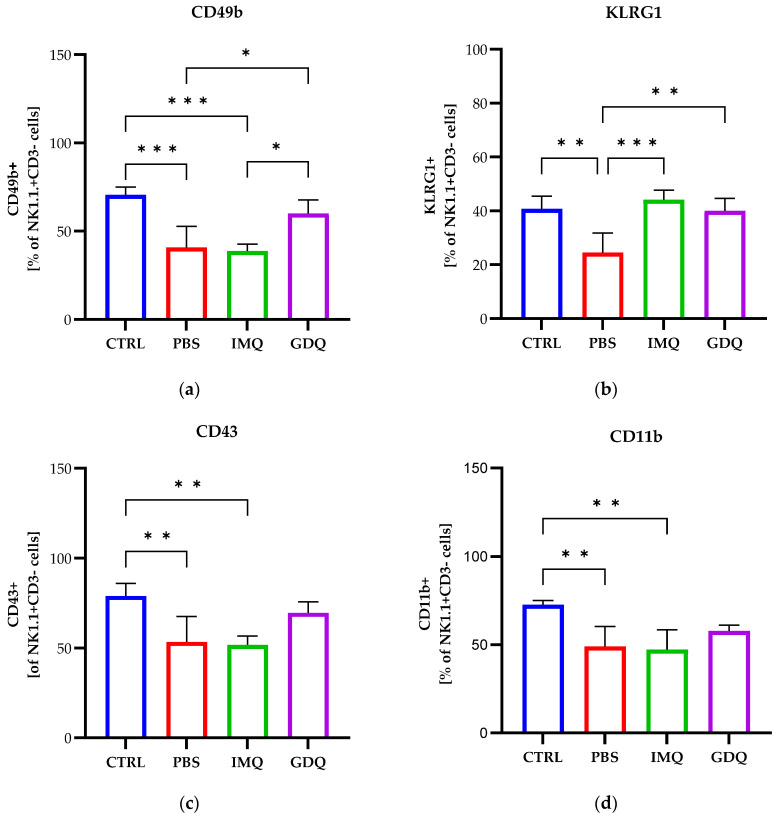

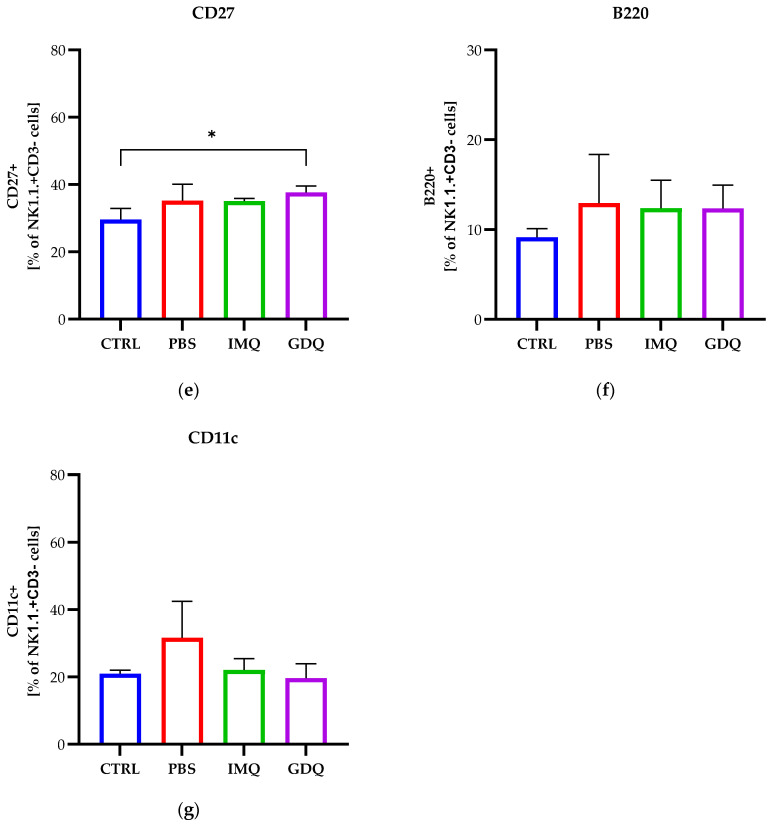
Spleen-derived NK cells isolated from C57BL/6 mice (CTRL, *n* = 5), melanoma-bearing mice (PBS, *n* = 5), and MbM intratumorally injected with imiquimod (IMQ, *n* = 5) or gardiquimod (GDQ, *n* = 4) were evaluated for the expression of the maturation receptors CD49b (**a**), KLRG1 (**b**), CD43 (**c**), CD11b (**d**), CD27 (**e**), B220 (**f**), or CD11c (**g**). One-way ANOVA with Tukey’s multiple comparison test was used to determine the statistical significance of differences between groups. Data are presented as mean ± SD; * *p* < 0.05, ** *p* < 0.01 and *** *p* < 0.001.

When monitoring the CD49b-positive population of NK cells, a reduced frequency was observed for the PBS and IMQ groups compared to healthy mice (*p* < 0.001), while for GDQ treatment the NK1.1+CD49b+ cells increased compared to the PBS (*p* < 0.05) and IMQ groups (*p* < 0.05) (Figure 8a). Also, the expression of the maturation marker KLRG1 was lower for the PBS group compared to the control group (*p* < 0.01), while TLR7/8 agonist treatment induced an increase in NK1.1+KLRG1+ cells compared to the PBS group (IMQ, *p* < 0.001; GDQ, *p* < 0.01), reaching percentages similar to those of healthy mice (Figure 8b). We also monitored the expression of CD43 and CD11b maturation markers, for which we observed a decrease in the case of the PBS and IMQ groups (*p* < 0.01), which was partially recovered by treatment with GDQ, but the values were still lower compared to the control group (Figure 8c,d).

Conversely, when monitoring the NK cells expressing CD27 or B220, a slight increase in these cell populations was observed for tumor-bearing mice compared to healthy mice, with a higher trend observed for the GDQ group (*p* < 0.05) with respect to CD27-positive NK cells (Figure 8e,f). TLR7/8 agonist treatment counteracted the increase in NK1.1+CD11c+ cells observed for the melanoma-bearing mice (PBS group), reaching values close to those of the healthy group for both the IMQ and GDQ groups of mice (Figure 8g).

## 4. Discussion

Although immunotherapy has made remarkable progress for cancer treatment, refractive response to therapy represents a major issue; therefore, sustained efforts are focused on finding new agents or therapeutic regimens that are effective in a wider range of tumors. In this regard, viable therapeutic targets are represented by Toll-like receptors, both as individual therapies or in combination with other therapies. Furthermore, a number of clinical trials have investigated Toll-like receptor agonists as single therapeutic agents or adjuvants in cancer treatment [49]. Although TLR agonists have been shown to reduce tumor burden in many types of cancer, their systemic application leads to side effects associated with aberrant systemic inflammatory response, including cytokine release syndrome [22].

In this study, we tested the efficacy of TLR7/8 agonists like imiquimod and gardiquimod, described above, as monotherapy in a mouse model of melanoma. Topical administration of imiquimod, ALDARA cream, on the established tumor significantly delayed the tumor growth, while intratumoral administration of imiquimod also inhibited tumor growth, however with less pronounced effects. Intratumoral administration of gardiquimod resulted in tumor growth reduction, with an increased potency compared to imiquimod. These results align with previous studies which evaluated combination therapy with a bone marrow-derived dendritic cell vaccine and imiquimod or gardiquimod in a B16-induced melanoma model [17].

We previously investigated the immunological changes in lymphocyte populations from peripheral blood and spleen induced by tumor formation in a mouse model of melanoma [27] and observed that the percentage of NK cells in the spleen of MbM was reduced compared to healthy mice and they displayed a different phenotype in population subsets. These observations led us to hypothesize that treatment with TLR agonists, particularly TLR7/8, might improve the phenotypic pattern of NK cells as potential therapy in melanoma. Although we observed delayed tumor growth, administration of TLR7/8 agonists did not significantly increase the percentage of NK cells for any of the chemicals, which were similar to those of tumor-bearing mice without treatment. A previous study also showed that treatment of RMA-S tumor-bearing mice with a small molecule agonist for TLR7 named SC1 did not result in an increase in NK cell number but induced significant NK cell activation [49].

Considering these observations, we further decided to evaluate the activation phenotype of NK cells since it may be that TLR agonists do not induce quantitative changes but rather an efficient NK activation. When imiquimod was administered topically, we observed that the percentage of B220+CD11c+NK1.1+ cells was still at high levels, similar to those in untreated mice, suggesting activation via TLR7-mediated signaling does not impair the NK activation phenotype. Moreover, our results showed that the percentage of the immature CD27-CD11b- subset and the early mature CD27+CD11b- subset was decreased for the imiquimod-treated group compared to MbM, while the percentage of the mature CD27+CD11b+ and CD27-CD11b+ subsets was increased, with the final stage of maturation value being similar to those of healthy mice. This implies that the TLR7 agonist leads to better activation and maturation of NK cells with higher capacity for cytokine secretion and cytotoxic activity, thus potentially explaining the tumor growth restriction observed for ALDARA-treated mice. Also, topical administration resulted in an increased expression of some markers for NK cell activation and maturation, such as CD69, NKp46, CD122, CD11b, CD43, KLRG1, and CD27, when compared with melanoma-bearing mice. All these data reflect a phenotypically activated profile of NK cells, which is consistent with our previous studies where we showed that topical administration of imiquimod (Aldara cream) induces NK cell activation [50,51].

On the other hand, intratumoral administration of imiquimod or gardiquimod decreased the percentage of B220+CD11c+NK1.1+ cells compared to untreated mice, with guardiquimod inducing changes very similar to healthy mice. Comparison of the distribution of NK cell subsets between healthy and melanoma-bearing mice, with or without treatment, showed a significant reduction in mature and an increase in immature NK cell subsets in the case of intratumoral imiquimod administration. A similar distribution pattern of NK cell subsets was also observed with gardiquimod treatment, with values very similar to the healthy group of mice. NK cells from mice with intratumoral treatment displayed a considerable decrease in the expression of the activating receptor NKp46 compared to healthy mice, while imiquimod treatment decreased the percentage of CD122-positive cells. CD69 receptor presented decreased values compared to the melanoma group, but the expression was slightly increased compared to healthy mice. The maturation markers CD49, CD43, and CD11b showed decreased values compared to healthy mice; in the case of gardiquimod treatment, the values were higher compared to both the untreated and imiquimod groups, with values similar to the healthy group.

Both treatment groups presented decreased CD11c expression compared to untreated mice, with values very similar to those of healthy mice. The expression of gp49R receptor was increased in both treatment groups compared to the control and melanoma groups. Increased values were also observed for CD27 and B220 compared to the control group, but not the melanoma group, while KLRG1 expression was higher than for the melanoma group, approaching that of healthy animals.

Based on the results from this model, we can propose that TLR agonists are able to induce phenotypic modulation of NK cells in a mouse model of melanoma. Furthermore, our results suggest that the route of administration of the treatment or the therapeutic regimen may lead to differences in the expression of various surface markers on NK cells and tumor growth restriction. This study reveals that treatment with gardiquimod or imiquimod delayed the growth of melanoma and induced a phenotypically activated profile of NK cells with possible effects in tumor control.

However, while our observation could provide insights into new therapeutic approaches, we have identified several limitations of the study mainly related to our observations being focused on extensively characterizing the phenotype of NK cells, with less emphasis on functional assessment of NK-mediated cytotoxicity, and to the relatively small number of animals per group. We acknowledge that further studies are necessary to thoroughly dissect the mechanisms by which imiquimod and gardiquimod enhance the antitumor capacity of NK cells and the relation between NK cells and other immune cells involved in tumor control. Nonetheless, our observations provide important evidence that the NK phenotype can be modulated by TLR7/8 agonists to restrict melanoma tumor growth.

In summary, imiquimod and particularly gardiquimod could serve as potent neoadjuvant, adjuvant, or complementary immunotherapeutic agents in melanoma therapy.

## 5. Conclusions

Topical administration of imiquimod, ALDARA cream, on established tumors delayed tumor growth, while intratumoral administration of imiquimod displayed a less pronounced effect. However, significantly delayed tumor growth was observed when gardaquimod was administered intratumorally. This study provides new perspectives on NK cell phenotype in solid tumors after TLR7/8 agonist administration, and our findings on NK cell dynamics during treatment with Toll-like receptor 7/8 agonists in melanoma could be used to establish new approaches in cancer immunotherapy. As future prospects, the potential of gardiquimod as an immune modifier in melanoma should be further exploited in combinatorial treatments, including as an adjuvant in melanoma-directed vaccine-based strategies. To assess the potential usefulness of this treatment, immunohistochemical studies of NK cell infiltration into the tumor could potentially contribute to selecting patients that would benefit most from this intervention.

## Data Availability

All data included in the article are available from the corresponding author upon reasonable request.

## Abbreviations

The following abbreviations are used in this manuscript:

TLRs	Toll-like receptors
NK	Natural killer
PAMPs	Pathogen-associated molecular patterns
PRR	Pattern recognition receptors
IL	Interleukin
APCs	Antigen-presenting cells
INF-γ	Interferon gamma
TNF-α	Tumor necrosis factor alpha

## References

[1] Naghavi M., Kyu H.H., Bhoomadevi A., Aalipour M.A., Aalruz H., Ababneh H.S., Abafita B.J., Abaraogu U.O., Abbafati C., Abbasi M. (2025). Global Burden of 292 Causes of Death in 204 Countries and Territories and 660 Subnational Locations, 1990–2023: A Systematic Analysis for the Global Burden of Disease Study 2023. Lancet.

[2] Manganelli M., Stabile G., Scharf C., Podo Brunetti A., Paolino G., Giuffrida R., Bigotto G.D., Damiano G., Mercuri S.R., Sallustio F. (2025). Skin Photodamage and Melanomagenesis: A Comprehensive Review. Cancers.

[3] Petit V., Raymond J., Alberti C., Pouteaux M., Gallagher S.J., Nguyen M.Q., Aplin A.E., Delmas V., Larue L. (2019). C57BL/6 Congenic Mouse NRAS^Q61K^ Melanoma Cell Lines Are Highly Sensitive to the Combination of Mek and Akt Inhibitors in Vitro and in Vivo. Pigment Cell Melanoma Res..

[4] Tellenbach F.L., Seiler L., Johnson M., Rehrauer H., Schukla P., Martinez-Gomez J., Stoffel C.I., Kamal A., Dummer R., Levesque M.P. (2025). Combination of the Novel RAF Dimer Inhibitor Brimarafenib with the MEK Inhibitor Mirdametinib Is Effective Against NRAS Mutant Melanoma. Pigment Cell Melanoma Res..

[5] Passarelli A., Mannavola F., Stucci L.S., Tucci M., Silvestris F. (2017). Immune System and Melanoma Biology: A Balance between Immunosurveillance and Immune Escape. Oncotarget.

[6] Noh J.-Y., Yoon S.R., Kim T.-D., Choi I., Jung H. (2020). Toll-Like Receptors in Natural Killer Cells and Their Application for Immunotherapy. J. Immunol. Res..

[7] Oth T., Habets T.H.P.M., Germeraad W.T.V., Zonneveld M.I., Bos G.M.J., Vanderlocht J. (2018). Pathogen Recognition by NK Cells Amplifies the Pro-Inflammatory Cytokine Production of Monocyte-Derived DC via IFN-γ. BMC Immunol..

[8] Gallardo-Zapata J., Maldonado-Bernal C. (2021). Role of Toll-like Receptors in Natural Killer Cell Function in Acute Lymphoblastic Leukemia (Review). Oncol. Lett..

[9] Gorski K.S., Waller E.L., Bjornton-Severson J., Hanten J.A., Riter C.L., Kieper W.C., Gorden K.B., Miller J.S., Vasilakos J.P., Tomai M.A. (2006). Distinct Indirect Pathways Govern Human NK-Cell Activation by TLR-7 and TLR-8 Agonists. Int. Immunol..

[10] Urban-Wojciuk Z., Khan M.M., Oyler B.L., Fåhraeus R., Marek-Trzonkowska N., Nita-Lazar A., Hupp T.R., Goodlett D.R. (2019). The Role of TLRs in Anti-Cancer Immunity and Tumor Rejection. Front. Immunol..

[11] Kauffman E.C., Liu H., Schwartz M.J., Scherr D.S. (2012). Toll-Like Receptor 7 Agonist Therapy with Imidazoquinoline Enhances Cancer Cell Death and Increases Lymphocytic Infiltration and Proinflammatory Cytokine Production in Established Tumors of a Renal Cell Carcinoma Mouse Model. J. Oncol..

[12] Xiong Z., Ohlfest J.R. (2011). Topical Imiquimod Has Therapeutic and Immunomodulatory Effects Against Intracranial Tumors. J. Immunother..

[13] Azin M., Ngo K.H., Hojanazarova J., Demehri S. (2023). Topical Calcipotriol Plus Imiquimod Immunotherapy for Nonkeratinocyte Skin Cancers. JID Innov..

[14] Chi H., Li C., Zhao F.S., Zhang L., Ng T.B., Jin G., Sha O. (2017). Anti-Tumor Activity of Toll-Like Receptor 7 Agonists. Front. Pharmacol..

[15] Vaienti S., Calzari P., Nazzaro G. (2023). Topical Treatment of Melanoma In Situ, Lentigo Maligna, and Lentigo Maligna Melanoma with Imiquimod Cream: A Systematic Review of the Literature. Dermatol. Ther..

[16] Hackstein H., Hagel N., Knoche A., Kranz S., Lohmeyer J., Von Wulffen W., Kershaw O., Gruber A.D., Bein G., Baal N. (2012). Skin TLR7 Triggering Promotes Accumulation of Respiratory Dendritic Cells and Natural Killer Cells. PLoS ONE.

[17] Ma F., Zhang J., Zhang J., Zhang C. (2010). The TLR7 Agonists Imiquimod and Gardiquimod Improve DC-Based Immunotherapy for Melanoma in Mice. Cell Mol. Immunol..

[18] Sun H., Li Y., Zhang P., Xing H., Zhao S., Song Y., Wan D., Yu J. (2022). Targeting Toll-like Receptor 7/8 for Immunotherapy: Recent Advances and Prospectives. Biomark. Res..

[19] Anwar M.A., Shah M., Kim J., Choi S. (2019). Recent Clinical Trends in Toll-like Receptor Targeting Therapeutics. Med. Res. Rev..

[20] Shah D., Comba A., Faisal S.M., Kadiyala P., Baker G.J., Alghamri M.S., Doherty R., Zamler D., Nuñez G., Castro M.G. (2021). A Novel miR1983-TLR7-IFNβ Circuit Licenses NK Cells to Kill Glioma Cells, and Is under the Control of Galectin-1. OncoImmunology.

[21] Aspord C., Tramcourt L., Leloup C., Molens J.-P., Leccia M.-T., Charles J., Plumas J. (2014). Imiquimod Inhibits Melanoma Development by Promoting pDC Cytotoxic Functions and Impeding Tumor Vascularization. J. Investig. Dermatol..

[22] Li Y., Chen Y., Tang Y., Yang T., Zhou P., Miao L., Chen H., Deng Y. (2025). Breaking the Barriers in Effective and Safe Toll-like Receptor Stimulation via Nano-Immunomodulators for Potent Cancer Immunotherapy. J. Control. Release.

[23] Hart O.M., Athie-Morales V., O’Connor G.M., Gardiner C.M. (2005). TLR7/8-Mediated Activation of Human NK Cells Results in Accessory Cell-Dependent IFN-γ Production. J. Immunol..

[24] Surcel M., Constantin C., Munteanu A.N., Costea D.A., Isvoranu G., Codrici E., Popescu I.D., Tănase C., Ibram A., Neagu M. (2023). Immune Portrayal of a New Therapy Targeting Microbiota in an Animal Model of Psoriasis. J. Pers. Med..

[25] Oya K., Nakamura Y., Zhenjie Z., Tanaka R., Okiyama N., Ichimura Y., Ishitsuka Y., Saito A., Kubota N., Watanabe R. (2021). Combination Treatment of Topical Imiquimod Plus Anti-PD-1 Antibody Exerts Significantly Potent Antitumor Effect. Cancers.

[26] Dumitru C.D., Antonysamy M.A., Gorski K.S., Johnson D.D., Reddy L.G., Lutterman J.L., Piri M.M., Proksch J., McGurran S.M., Egging E.A. (2009). NK1.1+ Cells Mediate the Antitumor Effects of a Dual Toll-like Receptor 7/8 Agonist in the Disseminated B16-F10 Melanoma Model. Cancer Immunol. Immunother..

[27] Isvoranu G., Surcel M., Huică R.-I., Munteanu A., Pirvu I., Ciotaru D., Constantin C., Bratu O., Neagu M., Ursaciuc C. (2019). Natural Killer Cell Monitoring in Cutaneous Melanoma—New Dynamic Biomarker. Oncol. Lett..

[28] Spits H., Lanier L.L. (2007). Natural Killer or Dendritic: What’s in a Name?. Immunity.

[29] Vosshenrich C.A.J., Lesjean-Pottier S., Hasan M., Richard-Le Goff O., Corcuff E., Mandelboim O., Di Santo J.P. (2007). CD11cloB220+ Interferon-Producing Killer Dendritic Cells Are Activated Natural Killer Cells. J. Exp. Med..

[30] Liu C.-F., Min X.-Y., Wang N., Wang J.-X., Ma N., Dong X., Zhang B., Wu W., Li Z.-F., Zhou W. (2017). Complement Receptor 3 Has Negative Impact on Tumor Surveillance through Suppression of Natural Killer Cell Function. Front. Immunol..

[31] Blasius A.L., Barchet W., Cella M., Colonna M. (2007). Development and Function of Murine B220+CD11c+NK1.1+ Cells Identify Them as a Subset of NK Cells. J. Exp. Med..

[32] Kim S., Iizuka K., Kang H.-S.P., Dokun A., French A.R., Greco S., Yokoyama W.M. (2002). In Vivo Developmental Stages in Murine Natural Killer Cell Maturation. Nat. Immunol..

[33] Yang F.C., Agematsu K., Nakazawa T., Mori T., Ito S., Kobata T., Morimoto C., Komiyama A. (1996). CD27/CD70 Interaction Directly Induces Natural Killer Cell Killing Activity. Immunology.

[34] Grant E.J., Nüssing S., Sant S., Clemens E.B., Kedzierska K. (2017). The Role of CD27 in Anti-Viral T-Cell Immunity. Curr. Opin. Virol..

[35] Tanaka T. (2016). Leukocyte Adhesion Molecules. Encyclopedia of Immunobiology.

[36] Chiossone L., Chaix J., Fuseri N., Roth C., Vivier E., Walzer T. (2009). Maturation of Mouse NK Cells Is a 4-Stage Developmental Program. Blood.

[37] Jin J., Fu B., Mei X., Yue T., Sun R., Tian Z., Wei H. (2013). CD11b−CD27− NK Cells Are Associated with the Progression of Lung Carcinoma. PLoS ONE.

[38] Borrego F., Robertson M.J., Ritz J., Peña J., Solana R. (1999). CD69 Is a Stimulatory Receptor for Natural Killer Cell and Its Cytotoxic Effect Is Blocked by CD94 Inhibitory Receptor. Immunology.

[39] Hu Z.-W., Sun W., Wen Y.-H., Ma R.-Q., Chen L., Chen W.-Q., Lei W.-B., Wen W.-P. (2022). CD69 and SBK1 as Potential Predictors of Responses to PD-1/PD-L1 Blockade Cancer Immunotherapy in Lung Cancer and Melanoma. Front. Immunol..

[40] Koyama-Nasu R., Wang Y., Hasegawa I., Endo Y., Nakayama T., Kimura M.Y. (2022). The Cellular and Molecular Basis of CD69 Function in Anti-Tumor Immunity. Int. Immunol..

[41] Glasner A., Ghadially H., Gur C., Stanietsky N., Tsukerman P., Enk J., Mandelboim O. (2012). Recognition and Prevention of Tumor Metastasis by the NK Receptor NKp46/NCR1. J. Immunol..

[42] Williams N.S., Klem J., Puzanov I.J., Sivakumar P.V., Schatzle J.D., Bennett M., Kumar V. (1998). Natural Killer Cell Differentiation: Insights from Knockout and Transgenic Mouse Models and in Vitro Systems. Immunol. Rev..

[43] Wu Y., Tian Z., Wei H. (2017). Developmental and Functional Control of Natural Killer Cells by Cytokines. Front. Immunol..

[44] Geiger T.L., Sun J.C. (2016). Development and Maturation of Natural Killer Cells. Curr. Opin. Immunol..

[45] Clinthorne J.F., Beli E., Duriancik D.M., Gardner E.M. (2013). NK Cell Maturation and Function in C57BL/6 Mice Are Altered by Caloric Restriction. J. Immunol..

[46] Wang L.L., Chu D.T., Dokun A.O., Yokoyama W.M. (2000). Inducible Expression of the gp49B Inhibitory Receptor on NK Cells. J. Immunol..

[47] Li M., Zhao X. (2024). Leukocyte Immunoglobulin-like Receptor B4 (LILRB4) in Acute Myeloid Leukemia: From Prognostic Biomarker to Immunotherapeutic Target. Chin. Med. J..

[48] Sharma N., Atolagbe O.T., Ge Z., Allison J.P. (2021). LILRB4 Suppresses Immunity in Solid Tumors and Is a Potential Target for Immunotherapy. J. Exp. Med..

[49] Wiedemann G.M., Jacobi S.J., Chaloupka M., Krächan A., Hamm S., Strobl S., Baumgartner R., Rothenfusser S., Duewell P., Endres S. (2016). A Novel TLR7 Agonist Reverses NK Cell Anergy and Cures RMA-S Lymphoma-Bearing Mice. OncoImmunology.

[50] Surcel M., Munteanu A., Huică R., Isvoranu G., Pirvu I., Constantin C., Bratu O., Căruntu C., Zaharescu I., Sima L. (2019). Reinforcing Involvement of NK Cells in Psoriasiform Dermatitis Animal Model. Exp. Ther. Med..

[51] Surcel M., Munteanu A., Isvoranu G., Ibram A., Caruntu C., Constantin C., Neagu M. (2021). Unconventional Therapy with IgY in a Psoriatic Mouse Model Targeting Gut Microbiome. J. Pers. Med..

